# Comparison of the efficacy of connective tissue massage and manual lymphatic drainage in patients with migraine: a randomized controlled trial

**DOI:** 10.22514/jofph.2025.054

**Published:** 2025-09-12

**Authors:** Betül Yıldırım Bulut, Özlem Çinar Özdemir

**Affiliations:** ^1^Department of Physical Therapy, Vocational School of Health Services, Siirt University, 56100 Siirt, Turkey; ^2^Department of Physical Therapy and Rehabilitation, Faculty of Health Sciences, Bolu Abant Izzet Baysal University, 14030 Bolu, Turkey

**Keywords:** Migraine, Manual lymph drainage, Massage, Headache, Quality of life, Neck pain

## Abstract

**Background**: This study aimed to evaluate and compare the effects of connective tissue massage (CTM) and manual lymphatic drainage (MLD) on neck pain, disability, quality of life, and pain thresholds in patients with migraine. **Methods**: A total of 40 migraine patients were randomly assigned to either the CTM or MLD group. Quality of life was assessed using the Short Form-36 (SF-36); pain sensitivity and perception were measured using an algometer, and neck pain was evaluated using the Neck Pain and Disability Scale (NPAD). Patients tracked their headaches in a pain diary for 15 days before and after the intervention. Both groups received 45 min of treatment twice a week for six weeks. **Results**: After treatment, the pain threshold increased significantly, whereas total medication use and the number of pain days decreased in both groups. However, while CTM led to a greater reduction in NPAD scores (*p *< 0.001), MLD was more effective in improving overall pain management and enhancing quality of life (*p* = 0.017). **Conclusions**: These findings suggest that CTM and MLD can help migraine patients, with MLD showing a stronger effect on pain relief and well-being, whereas CTM being more effective in reducing neck pain and disability. **Clinical Trial Registration**: ClinicalTrials.gov/NCT05976399.

## 1. Introduction

Migraine is a chronic condition affecting millions of people worldwide. It is characterized by severe unilateral headaches that are pulsating, autonomic nerve dysfunction, and relapsing neurological symptoms in some patients [1, 2]. While the exact pathophysiology of migraine remains unresolved, some studies have found that it is associated with mechanisms such as thalamocortical dysrhythmia, central sensitization, and activation of the trigeminovascular system. Natural processes such as menstruation, pregnancy and menopause in women lead to hormonal fluctuations [3]. Therefore, the prevalence of migraine is three times greater in women than in men after puberty [4]. Pain is generally localized to the posterior cervical region and the trapezius muscle, with neck pain accompanying migraine attacks in about 75% of cases [5]. Additionally, neck pain may trigger migraine attacks, and migraine and neck pain can sometimes occur together [6]. Despite the use of various invasive and non-invasive methods, such as botulinum toxin injections, nerve blocks, relaxation training, electrotherapy modalities, exercise, manipulation and mobilization techniques, cold application, massage and trigger point therapy, for treating migraine, existing evidence-based guidelines do not provide sufficient solutions for clinical decision-making and patient care [7, 8]. Connective tissue massage (CTM) is a special manipulative treatment method that includes various techniques that target superficial connective tissue and subcutaneous tissues, and differs from traditional massage in its physiological effects and the techniques used. The techniques applied in CTM allow the effectiveness of the treatment to extend beyond the local stimulation area, producing effects on distant organs as well [9]. A preventive approach used for migraine is manual lymphatic drainage (MLD). Besides reducing the hyperexcitability of the central nervous system, the effects of MLD on the sympathetic and parasympathetic nervous systems resemble those of relaxation therapy, resulting in overall relaxation [10, 11]. Some studies have shown that the meningeal lymphatic system is located in the brain and functions as a novel drainage system for cerebrospinal fluid [12, 13]. These findings further support the role of MLD in migraine management by highlighting its effect on cerebrospinal fluid and its effect on brain-related physiological processes [14]. Several studies have examined the effects of MLD on pain management and treatment in conditions such as carpal tunnel syndrome, anterior cruciate ligament injuries, chest pain and fullness after cesarean section, and fibromyalgia [15, 16, 17, 18]. However, studies evaluating the efficacy of MLD for migraine patients are scarce. In this randomized controlled trial, we improved the quality of life of migraine patients, decreased their pain, and increased the awareness of health professionals in this field.

## 2. Materials and methods

### 2.1 Study design

We conducted a randomized controlled study involving volunteers who were diagnosed with migraine, resided in Burdur Province, and were registered at Burdur Family Health Centers. Participants were recruited, and data were collected between January 2020 and December 2020. All participants were informed about the requirements of the study, provided written informed consent, and were randomized using Quickcalcs on the GraphPad website into two groups: the CTM group (n = 20) and the MLD group (n = 20). All participants were assessed before and after treatment for six weeks. The participants in the MLD group received MLD for six weeks, whereas the participants in the CTM group underwent CTM for the same duration. The participants in both groups continued the pharmacological treatments prescribed by their doctors during the study period. All assessments were performed in person before and after the six-week follow-up.

This study was approved by the Ethics Committee of Izmir Democracy University Clinical Research (approval number: 2019/06-04). Necessary permission was obtained from the Burdur Provincial Health Directorate to contact patients diagnosed with migraine. This clinical study (ClinicalTrials.gov/NCT05976399) was performed in compliance with the Declaration of Helsinki guidelines. Participants who met the inclusion criteria were selected, and after being informed about the study, written and verbal consent were obtained. All participants were informed about the requirements of the study.

### 2.2 Study population

The sample size was determined using the G*Power 3.1.9 software package (Heinrich Heine University, Düsseldorf, NRW, Germany). The effect size was determined as *dz* = 1.149, following the methods described by Kesikburun *et al*. [19]. The minimum sample size, which was calculated with 95% statistical power and a 0.05 margin of error, was 24 patients: 12 in the CTM group and 12 in the MLD group. In total, 50 patients were initially planned to be included in the study; however, following interviews and evaluations with the participants, six patients refused to participate, and four patients were excluded from the study because they did not meet the inclusion criteria. Finally, 40 patients were included in the study (Fig. 1).

**Fig. 1.** The flowchart of the study.

The inclusion criteria were as follows: literate participants; those diagnosed with migraine according to the criteria of the International Headache Society by a neurologist; migraine patients who were between 18 and 65 years old, volunteered to participate, and provided signed informed consent; patients with intact cognitive functions; participants who marked a pain intensity of over 40 mm on the visual analog scale (VAS); and patients who experienced headaches (without allodynia) less than 15 days per month. The exclusion criteria included patients with contraindications for MLD in the general or neck region; patients with a history of neuromuscular disorders; those with communication difficulties that hinder evaluation; patients with headaches lasting more than 15 days per month; participants diagnosed with other types of headaches, such as tension-type or cervicogenic headaches; fibromyalgia or myofascial pain syndrome; patients with a history of whiplash or similar traumas or severe depression symptoms, as indicated by the Beck Depression Inventory; patients with congenital musculoskeletal abnormalities; those who received non-pharmacological treatments for migraine (*e.g.*, acupuncture or dry needling); and patients who had been undergoing a neck physiotherapy program for at least three months.

### 2.3 Intervention

An initial assessment was conducted to determine patient eligibility. After the assessment, all participants were assigned to either the CTM (n = 20) or MLD (n = 20) groups by simple randomization to be included in a physiotherapy program. Pain diaries were provided to all patients two weeks before the intervention and after treatment to record pain-related characteristics. The participants were instructed to fill out their pain diary regularly two weeks before the treatment. The duration of the treatment was 45 min twice a week for six weeks. After the treatment, the patients were monitored for 15 days, and a pain diary was used to record pain-related characteristics. To minimize therapist-related variability and ensure the standardization of interventions, all treatments were administered by a single physiotherapist with certified expertise and specialized training in the respective techniques. This approach eliminated inter-therapist inconsistencies and improved the methodological rigor and reliability of the treatment process.

#### 2.3.1 MLD group

Patients who completed their pain diaries and submitted a consent form were assessed before treatment. They were instructed to lie on their back with the neck area exposed, ensuring that patient privacy was maintained. A fundamental treatment technique, known as hand stroke, is planned based on the physiology and anatomy of the lymphatic system. The working phase during hand strokes was applied for 1 s. MLD was applied to the lateral neck and face area in 5–7 repetitions to ensure an adequate response to the treatment. The sessions were conducted twice a week for six weeks (n = 12 sessions), with each session lasting 45 min. MLD applications were performed by a physiotherapist who had completed the 135-h training program provided by the Academy of Lymphatic Studies (ACOLS) and who held the title of Certified Lymphedema Therapist (CLT). The treatment protocol was based on the Vodder technique and current lymphedema management guidelines [20].

#### 2.3.2 CTM group

Patients who had completed their pain diaries and submitted signed consent forms were evaluated before treatment. The participants were instructed to sit upright on a chair with their sacral, lumbar, and scapular regions exposed. The treatment started in the sacral region, referred to as the “basic region”, and then later involved the scapular, interscapular and lower thoracic regions. The manipulations in the interscapular region were more intense than those in other areas. After these regions were treated, the cervical, clavicular and facial areas were treated. For facial treatments, patients were positioned in a supine position on a stretcher. Each area was treated three times on the right and left sides. During CTM, short and long manipulations were performed based on the characteristics of each region. Patient privacy was ensured through proper positioning, and the room temperature was maintained at a level that was comfortable for the patients. A total of 12 sessions were conducted twice a week over six weeks. CTM was performed by the same physiotherapist who applied the treatments to the MLD group. During the applications, the most recent sources of CTM techniques were used [21].

### 2.4 Measurements

A patient information form was used to collect the general clinical information of patients about migraine and their sociodemographic data (such as age, weight and height), contact information and education status. Additionally, general clinical details, such as age at onset of migraine, frequency, severity, duration and symptoms accompanying migraine, were recorded.

#### 2.4.1 Neck pain and disability scale (NPAD)

The NPAD scale was created by Wheeler *et al*. [22], and the validity and reliability of the Turkish version were assessed by Biçer *et al*. [23]. The scale comprises 20 questions that examine the relationship between neck pain severity and social, functional and emotional well-being. Each item is scored on a scale of 0–5, where a higher score indicates greater disability in the patient.

#### 2.4.2 Visual analog scale (VAS)

This scale consists of a horizontal or vertical line 100 mm long, where “0” represents no pain and “10” represents the most severe pain [24]. The patients were asked to rate their pain by marking a point on the line between the extremes (“0—No pain” and “10—Unbearable pain”). The distance from the “0” point to the marked point was measured in millimeters and recorded as the pain intensity.

#### 2.4.3 Pain diary

The pain diary is used to plan and evaluate the effectiveness of treatment. Patients are asked to document details such as the onset, intensity, location of pain, factors that exacerbate or alleviate it, and name and dosage of any medication taken (if applicable) in a diary [25]. A pain diary was given to all the 40 migraine patients two weeks before starting the treatment. They were instructed to record the intensity and duration of pain, any accompanying symptoms and the name and dosage of medications taken during a headache in the diary. After six weeks of treatment, the patients were given the diary again to document the same information.

#### 2.4.4 Pressure algometer (Dolorimeter)

Fischer [26] developed a pressure algometer to evaluate the perception of pressure and measure pain sensitivity. Before using the dolorimeter, patients were informed about the measurement procedure. They were instructed to indicate by saying “stop” as soon as they experienced discomfort or pain. The most commonly affected muscles in patients with migraine are the sternocleidomastoid (SCM) and temporalis anterior neck and shoulder muscles. Sensitivity occurs in these muscles and various structures that are located here during common migraine attacks [27]. Measurements were taken using the dolorimeter in 12 regions (six on the right and six on the left). The following six muscles were selected: the temporalis anterior, SCM, scalenus anterior, suboccipital, levator scapulae and upper trapezius. Before the measurement was taken, the muscles were palpated and the target area was marked. The pressure was progressively increased at a rate of 1 kg/s. Measurements were made at 30-second intervals at each marked region, and the mean values were recorded.

#### 2.4.5 Short form-36 (SF-36)

The SF-36 scale was designed by Ware and Sherbourne [28], and its Turkish version was validated by Koçyigit *et al*. [29]. It is widely used to evaluate quality of life. This form consists of 36 questions that evaluate eight distinct domains: physical functioning, social functioning, limitations in roles due to physical health issues, limitations in roles due to emotional health problems, mental well-being, overall health perception, pain, and energy/vitality. Each domain is assessed independently, and a high score in any domain reflects a higher quality of life in that specific area.

### 2.5 Statistical analysis

Statistical analyses were performed using the IBM SPSS 23.0 software package (IBM Corp., Armonk, NY, USA). Descriptive data were reported as frequencies and percentages (n, %) for categorical variables and as the mean ± standard deviation or median (range) for continuous variables. Relationships between categorical variables were assessed by conducting Fisher’s exact test and Pearson’s Chi-square test where applicable. Pairwise comparisons were conducted with Bonferroni correction. The Shapiro-Wilk test was performed to determine whether the data followed a normal distribution. Student’s *t*-test was conducted for normally distributed data, whereas the Mann-Whitney U test was conducted for non-normally distributed data. Variations in measurement values and scale scores before and after the intervention were analyzed by conducting the Wilcoxon signed rank test for data that did not follow a normal distribution, and the paired samples *t*-test for data that followed a normal distribution. Effect sizes for pretreatment and post-treatment comparisons were calculated using Cohen’s *d* statistic.

## 3. Results

Of the 40 participants, 36 (90%) were females and four (10%) were males. Age, sex, height, weight and body mass index (BMI) were not significantly different between the CTM and MLD groups (Table 1).

**Table 1.** Patient demographics by study groups.

Variables		All patients (n = 40)	CTM (n = 20)	MLD (n = 20)	Value/*p*
Age (yr)		38.22 ± 11.99	37.40 ± 13.22	39.05 ± 10.91	*t* = −0.430 *p* = 0.669
Gender					
	Female	36 (90%)	19 (95%)	17 (85%)	*p* = 0.605
	Male	4 (10%)	1 (5%)	3 (15%)	
Height (cm)		1.65 ± 0.07	1.64 ± 0.07	1.65 ± 0.07	*t* = −0.181 *p *= 0.858
Weight (kg)		67.55 ± 11.06	67.30 ± 10.80	67.80 ± 11.59	*t* = −0.141 *p* = 0.889
BMI (kg/m^2^)		24.88 ± 3.53	24.83 ± 3.35	24.93 ± 3.79	*t* = −0.087 *p* = 0.931

*p < 0.05; MLD: Manual lymph drainage; CTM: Connective tissue massage; BMI: Body mass index. The results are shown as the mean ± standard deviation or n (%). Student’s t-test; Fisher’s Exact test.*

The mean NPAD scores before and after treatment are shown in Table 2. A significant decrease in NPAD scores was observed in the CTM and MLD groups post-treatment, indicating reduced neck pain-related disability. Additionally, while no significant difference was found between the two groups in terms of pain duration, number of headache days, or medication use, all parameters significantly improved within each group after treatment. These findings suggested that CTM and MLD contribute to reducing pain duration and medication dependency in migraine patients. However, the magnitude of improvement was similar in both groups, highlighting that their effectiveness in pain management was similar.

**Table 2.** Comparison of the neck pain and pain diary parameters among groups.

		All patients (n = 40)	Cohen *d*	CTM (n = 20)	Cohen *d*	MLD (n = 20)	Cohen *d*	Value/*p*
NPAD								
	Pretreatment	25.5 (4–81)		33.5 (7–81)		21.5 (4–61)		U = 155 *p* = 0.231
	Post-treatment	22.5 (2–68)		29.5 (2–68)		20.5 (4–56)		U = 167 *p* = 0.383
	Value/*p*	*Z* = −4.399 ***p* < 0.001**		*Z* = −3.731 ***p* < 0.001**		*Z* = −2.386 ***p* = 0.017**		
Headache (d)								
	Pretreatment	3.5 (0–9)		4 (0–9)		3 (0–6)		U = 163.5 *p* = 0.327
	Post-treatment	2 (0–9)		3 (0–9)		2 (0–6)		U = 173 *p* = 0.478
	Value/*p*	*Z* = −3.820 ***p* < 0.001**	*d* = 0.86	*Z* = −2.520 ***p* = 0.012**	*d* = 0.75	*Z* = −2.994 ***p* = 0.003**	*d* = 1.0	
Duration of the pain (h)								
	Pretreatment	24.5 (0–90)		23 (0–90)		24.5 (0–48)		U = 180.5 *p* = 0.602
	Post-treatment	19 (0–72)		24 (0–72)		18 (0–48)		U = 171.5 *p* = 0.445
	Value/*p*	*Z* = −2.309 ***p* = 0.021**	*d* = 0.45	*Z* = −1.468 *p* = 0.142		*Z* = −1.801 *p* = 0.072		
Number of medications								
	Pretreatment	4 (0–13)		4.5 (0–13)		3 (0–8)		U = 144.5 *p* = 0.134
	Post-treatment	2 (0–8)		3 (0–8)		2 (0–5)		U = 153 *p* = 0.211
	Value/*p*	*Z* = −4.767 ***p* < 0.001**	*d* = 1.0	*Z* = −3.440 ***p* = 0.001**	*d* = 1.0	*Z* = −3.351 ***p* = 0.001**	*d* = 1.0	

*Bold values indicate statistically significant differences, p < 0.05. NPAD: Neck Pain Disability Score; MLD: Manual lymph drainage; CTM: Connective tissue massage. Results are shown as median (min–max) values. Mann-Whitney U test; Wilcoxon Signed Ranks test.*

The comparison of pain algometry parameters before and after treatment in the CTM and MLD groups is presented in Tables 3a and 3b. A significant increase in pressure pain thresholds (PPTs) was observed in multiple muscle groups after treatment in both groups, indicating reduced pain sensitivity (*p* < 0.001). Improvements were observed in the anterior scalene, trapezius and suboccipital muscles (*p* < 0.001 for most parameters; Table 3a). While no significant difference was found between the CTM and MLD groups in terms of post-treatment values (*e.g.*, right anterior scalene *p* = 0.529), both groups showed significant intragroup improvements (*p* < 0.001), suggesting that both techniques can effectively increase PPTs. Similarly, PPTs increased considerably in the temporalis, levator scapula and SCM muscles following treatment (*p* < 0.05 for all comparisons; Table 3b). Although pretreatment comparisons revealed some differences between the groups, the post-treatment results showed that CTM and MLD effectively decreased pain sensitivity. These findings highlighted the efficacy of both CTM and MLD in improving PPTs in migraine patients. However, the similarity in post-treatment values suggested that both techniques have similar benefits in pain management.

**Table 3a.** Comparison of pain algometry parameters according to the study groups.

Variables		All patients (n = 40)	Cohen *d*	CTM (n = 20)	Cohen *d*	MLD (n = 20)	Cohen *d*	Value/*p*
Right anterior scalene								
	Pretreatment	4.2 (1.43–13.26)		3.96 (1.46–8.48)		4.92 (1.43–13.26)		U = 238.5 *p* = 0.301
	Post-treatment	4.3 (1.5–13.27)		4.01 (2.07–8.63)		5.4 (1.5–13.27)		U = 224 *p* = 0.529
	Value/*p*	*Z* = −5.109 ***p* < 0.001**	*d* = 0.80	*Z* = −3.342 ***p* = 0.001**	*d* = 0.73	*Z* = −3.921 ***p* < 0.001**	*d* = 0.96	
Left anterior scalene								
	Pretreatment	4.2 (1.38–13.84)		3.84 (1.77–8.64)		4.74 (1.38–13.84)		U = 243 *p* = 0.253
	Post-treatment	4.27 (1.39–13.86)		3.97 (2.04–8.7)		5.28 (1.39–13.86)		U = 233 *p* = 0.383
	Value/*p*	*Z* = −5.304 ***p* < 0.001**	*d* = 0.78	*Z* = −3.724 ***p* < 0.001**	*d* = 0.78	*Z* = −3.827 ***p* < 0.001**	*d* = 0.77	
Right trapezius								
	Pretreatment	4.31 ± 1.75		3.72 ± 1.58		4.9 ± 1.75		*t* = −2.216 ***p* = 0.033**
	Post-treatment	4.6 ± 1.66		4.08 ± 1.49		5.13 ± 1.69		*t* = −2.074 ***p* = 0.045**
	Value/*p*	*t* = −5.915 ***p* < 0.001**	*d* = 0.94	*t* = −4.242 ***p* < 0.001**	*d* = 0.95	*t* = −4.469 ***p* < 0.001**	*d* = 0.99	
Left trapezius								
	Pretreatment	4.39 ± 1.78		3.87 ± 1.68		4.91 ± 1.76		*t* = −1.905 *p* = 0.064
	Post-treatment	4.68 ± 1.73		4.2 ± 1.61		5.15 ± 1.76		*t* = −1.765 *p* = 0.086
	Value/*p*	*t* = −5.533 ***p* < 0.001**	*d* = 0.88	*t* = −4.108 ***p* = 0.001**	*d* = 0.92	*t* = −3.711 ***p* = 0.001**	*d* = 0.83	
Right suboccipital								
	Pretreatment	3.17 ± 1.36		2.75 ± 1.3		3.6 ± 1.33		*t* = −2.038 ***p* = 0.049**
	Post-treatment	3.45 ± 1.38		3.08 ± 1.34		3.82 ± 1.34		*t* = −1.755 *p* = 0.087
	Value/*p*	*t* = −4.743 ***p* < 0.001**	*d* = 0.75	*t* = −3.307 ***p* = 0.004**	*d* = 0.74	*t* = −3.605 ***p* = 0.002**	*d* = 0.81	
Left suboccipital								
	Pretreatment	3.12 ± 1.43		2.82 ± 1.41		3.41 ± 1.42		*t* = −1.315 *p* = 0.196
	Post-treatment	3.38 ± 1.41		3.15 ± 1.39		3.61 ± 1.44		*t* = −1.041 *p* = 0.304
	Value/*p*	*t* = −5.700 ***p* < 0.001**	*d* = 0.90	*t* = −4.104 ***p* = 0.001**	*d* = 0.92	*t* = −4.395 ***p* < 0.001**	*d* = 0.98	

*Bold values indicate statistically significant differences, p < 0.05. MLD: Manual lymph drainage; CTM: Connective tissue massage. Results are shown as mean ± standard deviation or median (min–max) values. Mann-Whitney U test; Student’s t-test; Wilcoxon Signed Ranks test; Paired Samples t-test.*

**Table 3b.** Comparison of pain algometry parameters according to the study groups.

Variables		All patients (n = 40)	Cohen *d*	CTM (n = 20)	Cohen *d*	MLD (n = 20)	Cohen *d*	Value/*p*
Right temporalis								
	Pretreatment	2.48 ± 1.15		2.2 ± 1.12		2.76 ± 1.15		*t* = −1.583 *p *= 0.122
	Post-treatment	2.81 ± 1.07		2.6 ± 1.03		3.02 ± 1.09		*t* = −1.258 *p *= 0.216
	Value/*p*	*t* = −5.934 ***p* < 0.001**	*d* = 0.94	*t* = −5.040 ***p* < 0.001**	*d* = 1.12	*t* = −3.392 ***p* = 0.003**	*d* = 0.76	
Left temporalis								
	Pretreatment	2.45 ± 1.19		2.2 ± 1.2		2.7 ± 1.15		*t* = −1.340 *p *= 0.188
	Post-treatment	2.76 ± 1.09		2.57 ± 1.1		2.95 ± 1.07		*t* = −1.082 *p *= 0.286
	Value/*p*	*t* = −6.296 ***p* < 0.001**	*d* = 0.99	*t* = −4.402 ***p* < 0.001**	*d* = 0.98	*t* = −5.083 ***p* < 0.001**	*d* = 114	
Right levator scapula								
	Pretreatment	2.08 ± 1.09		1.8 ± 0.9		2.36 ± 1.21		*t* = −1.667 *p *= 0.104
	Post-treatment	2.39 ± 1.06		2.19 ± 0.83		2.58 ± 1.24		*t* = −1.191 *p *= 0.242
	Value/*p*	*t* = −4.954 ***p* < 0.001**	*d* = 0.78	*t* = −3.375 ***p* = 0.003**	*d* = 0.76	*t* = −5.422 ***p* < 0.001**	*d* = 1.21	
Left levator scapula								
	Pretreatment	2.07 ± 1.06		1.81 ± 0.85		2.32 ± 1.2		*t* = −1.559 *p *= 0.127
	Post-treatment	2.32 ± 1.01		2.1 ± 0.76		2.53 ± 1.19		*t* = −1.365 *p *= 0.182
	Value/*p*	*t* = −5.286 ***p* < 0.001**	*d* = 0.84	*t* = −3.450 ***p* = 0.003**	*d* = 0.77	*t* = −4.750 ***p* < 0.001**	*d* = 1.06	
Right SCM								
	Pretreatment	2.58 ± 1.28		2.18 ± 1.16		2.98 ± 1.29		*t* = −2.055 ***p* = 0.047**
	Post-treatment	2.94 ± 1.25		2.57 ± 1.14		3.31 ± 1.28		*t* = −1.923 *p *= 0.062
	Value/*p*	*t* = −5.530 ***p* < 0.001**	*d* = 0.87	*t* = −3.822 ***p* = 0.001**	*d* = 0.86	*t* = −3.976 ***p* = 0.001**	*d* = 0.89	
Left SCM								
	Pretreatment	2.57 ± 1.24		2.24 ± 1.1		2.89 ± 1.32		*t* = −1.714 *p *= 0.095
	Post-treatment	2.92 ± 1.22		2.66 ± 1.07		3.18 ± 1.33		*t* = −1.363 *p *= 0.181
	Value/*p*	*t* = −4.457 ***p* < 0.001**	*d* = 0.71	*t* = −3.361 ***p* = 0.003**	*d* = 0.75	*t* = −2.913 ***p* = 0.009**	*d* = 0.65	

*Bold values indicate statistically significant differences, p < 0.05. MLD: Manual lymph drainage; CTM: Connective tissue massage; SCM: Sternocleidomastoid. Results are shown as mean ± standard deviation or median (min–max) values. Mann-Whitney U test; Student’s t-test; Wilcoxon Signed Ranks test; Paired Samples t-test.*

After treatment, significant improvements were observed in the energy/vitality (CTM: *p* = 0.001; MLD: *p* < 0.001), mental health (CTM: *p* = 0.019; MLD: *p* = 0.029), and social functioning (CTM: *p* = 0.006; MLD: *p* = 0.003) subscale scores in both the CTM and MLD groups. However, in the CTM group, the mean scores for the physical role (*p* = 0.083) and emotional role (*p* = 0.257) subscales were not significantly different between the pretreatment and post-treatment measurements, whereas these scores significantly improved after treatment in the MLD group (physical role: *p* = 0.046; emotional role: *p* = 0.038). In both groups, the SF-36 scores for pain (CTM: *p* < 0.001; MLD: *p* < 0.001), the total score (CTM: *p* < 0.001; MLD: *p* < 0.001), and general health perception (CTM: *p* = 0.018; MLD: *p* < 0.001) significantly increased after treatment (Table 4).

**Table 4.** Comparison of quality of life among study groups.

Sub-dimensions of SF-36		All patients (n = 40)	Cohen *d*	CTM (n = 20)	Cohen *d*	MLD (n = 20)	Cohen *d*	Value/*p*
Physical functioning								
	Pretreatment	80 (50–100)		80 (60–100)		82.5 (50–100)		U = 171 *p *= 0.445
	Post-treatment	85 (50–100)		82.5 (60–100)		85 (50–100)		U = 170 *p *= 0.429
	Value/*p*	*Z* = −1.857 *p *= 0.063		*Z* = −1.342 *p *= 0.180		*Z* = −1.342 *p *= 0.180		
Role limitations due to physical problems								
	Pretreatment	75 (0–100)		75 (0–100)		75 (25–100)		U = 224.5 *p *= 0.512
	Post-treatment	75 (0–100)		75 (0–100)		75 (25–100)		U = 229 *p *= 0.445
	Value/*p*	*Z* = −2.646 ***p* = 0.008**	*d* = 1.0	*Z* = −1.732 *p *= 0.083		*Z* = −2.000 ***p* = 0.046**	*d* = 0.49	
Role limitations due to emotional problems								
	Pretreatment	66.66 (0–100)		66.66 (33.33–100)		66.66 (0–100)		U = 205 *p *= 0.904
	Post-treatment	66.66 (0–100)		66.66 (33.33–100)		83.33 (0–100)		U = 225 *p *= 0.512
	Value/*p*	*Z* = −2.373 ***p* = 0.018**	*d* = 0.87	*Z* = −1.134 *p *= 0.257		*Z* = −2.070 ***p* = 0.038**	*d* = 0.56	
Energy/vitality								
	Pretreatment	46.63 ± 22.08		45.5 ± 20.89		47.75 ± 23.7		*t* = −0.318 *p *= 0.752
	Post-treatment	53.63 ± 19.84		52.5 ± 19.9		54.75 ± 20.23		*t* = −0.355 *p *= 0.725
	Value/*p*	*t* = −6.198 ***p* < 0.001**	*d* = 0.98	*t* = −3.907 ***p* = 0.001**	*d* = 0.88	*t* = −4.918 ***p* < 0.001**	*d* = 1.10	
Mental health								
	Pretreatment	62.7 ± 18.01		62.4 ± 19.27		63 ± 17.16		*t* = −0.587 *p *= 0.560
	Post-treatment	67.7 ± 14.89		68.4 ± 14.45		67 ± 15.67		*t* = −0.692 *p *= 0.493
	Value/*p*	*t* = −3.478 ***p* = 0.001**	*d* = 0.55	*t* = −2.555 ***p* = 0.019**	*d* = 0.57	*t* = −2.364 ***p* = 0.029**	*d* = 0.53	
Social functioning								
	Pretreatment	63.69 ± 18.7		64.25 ± 19.5		63.13 ± 18.35		*t* = 0.188 *p *= 0.852
	Post-treatment	73.06 ± 16.1		73.63 ± 17.06		72.5 ± 15.5		*t* = 0.218 *p *= 0.828
	Value/*p*	*t* = −4.685 ***p* < 0.001**	*d* = 0.74	*t* = −3.101 ***p* = 0.006**	*d* = 0.70	*t* = −3.470 ***p* = 0.003**	*d* = 0.77	
Pain								
	Baseline	51 ± 21.53		49.63 ± 22.69		52.38 ± 20.8		*t* = −0.400 *p *= 0.692
	Post-treatment	66.75 ± 19.02		65.13 ± 19.39		68.37 ± 19.01		*t* = −0.535 *p *= 0.596
	Value/*p*	*t* = −8.444 ***p* < 0.001**	*d* = 1.34	*t* = −5.754 ***p* < 0.001**	*d* = 1.29	*t* = −6.039 ***p* < 0.001**	*d* = 1.35	
The general perception of health								
	Baseline	54.92 ± 16.92		54.6 ± 17.82		55.25 ± 16.42		*t* = −0.120 *p *= 0.905
	Post-treatment	61.5 ± 16.42		60.5 ± 19.26		62.5 ± 13.43		*t* = −0.381 *p *= 0.705
	Value/*p*	*t* = −4.926 ***p* < 0.001**	*d* = 0.78	*t* = −2.580 ***p* = 0.018**	*d* = 0.58	*t* = −5.081 ***p* < 0.001**	*d* = 1.14	
Total Score								
	Baseline	2313.5 ± 462.79		2317.25 ± 373.01		2309.75 ± 548.14		*t* = 0.051 *p *= 0.960
	Post-treatment	2506.5 ± 438.89		2504.5 ± 376.03		2508.5 ± 503.96		*t* = −0.028 *p *= 0.977
	Value/*p*	*t* = −9.149 ***p* < 0.001**	*d* = 1.45	*t* = −5.766 ***p* < 0.001**	*d* = 1.29	*t* = −7.169 ***p* < 0.001**	*d* = 1.60	

*Bold values indicate statistically significant differences, p < 0.05. SF-36: Short Form-36; MLD: Manual lymph drainage; CTM: Connective tissue massage. Results are shown as mean ± standard deviation or median (min–max) values. Mann-Whitney U test; Student’s t-test; Wilcoxon Signed Ranks test; Paired Samples t-test.*

## 4. Discussion

Migraine is one of the most common neurological disorders and greatly affects the quality of life of individuals by increasing disability. Various treatment options can be used to manage migraine. CTM and MLD are among these treatment options [7]. Despite the high prevalence of migraine, there is limited research on the effects of migraine and MLD on migraine. This is the first study in Turkey to examine the effects of MLD on individuals with migraine. Although CTM and MLD applications had similar effects on pain, quality of life, and PPT levels in individuals with migraine, CTM application was more effective in terms of reducing neck disability scores. The findings of this study may increase awareness of the use of different physiotherapy methods for the rehabilitation of people suffering from migraine.

The symptoms of migraine may include pain and hypersensitivity with muscle and joint palpation in the neck and facial regions, restriction of cervical mobility, and forward head posture. It is still unclear whether neck pain is part of the prodromal (onset) symptoms of migraine and whether it is part of a migraine attack or acts as a trigger. Musculoskeletal disorders commonly occur in migraine patients, and neck pain is associated with additional disabilities; however, neck pain is also associated with more frequent migraine attacks and a poor prognosis after pharmacological treatment. Therefore, alleviating pain and disability in the neck can help patients cope with migraine pain [30].

In this study, participants with migraine were evaluated before and after treatment to determine the effect of their planned treatment on neck pain. The analysis revealed that both applications were effective in reducing neck pain and related disabilities, but the effect of CTM was greater than that of MLD. While MLD is a superficial application, CTM stimulates connective tissues in deeper tissues via its mechanical effects. Additionally, patients generally believe that deep touches may be more effective in applications [31]. This may explain the observed more effective results from the CTM application in our study.

Individuals with migraine exhibit altered neuromuscular function in the neck muscles; this is probably due to sensitization of trigeminal nociceptive pathways. Pain signals from the cervical muscles and dura mater converge at second-order neurons within the trigeminocervical complex, which may play a role in the initiation and maintenance of migraine headaches via cervical pain signals. Moreover, myofascial trigger points in muscles such as the suboccipital, trapezius, SCM, temporal and levator scapula are more common in migraine patients. This not only exacerbates headache symptoms but also decreases the functional mobility of these muscles [32, 33]. In support of these findings, a cross-sectional observational study evaluated the PPT in individuals with episodic and chronic migraine. The study revealed that both groups had significantly lower PPTs in trigeminal regions (*e.g.*, temporal and suboccipital muscles) and extra-trigeminal regions (*e.g.*, trapezius and tensor fasciae latae muscles) than did healthy individuals. Additionally, individuals with chronic migraine were found to have lower PPTs in the trigeminal regions, indicating greater pain sensitivity. These findings emphasized the role of cervical muscle sensitivity in the pathophysiology of migraine and highlighted the importance of addressing musculoskeletal disorders in the treatment of migraine [34]. In a study by Kurt and Turhan, 40 female patients diagnosed with chronic migraine were divided into two groups: the first group received superficial proprioceptive neuromuscular facilitation (PNF) techniques, whereas the second group received CTM treatment. Both groups participated in sessions of about 20 min three times a week for six weeks. PPT values were assessed using an algometer at seven different trigger points (m. occipitalis, m. trapezius, m. splenius cervicis, m. temporalis, m. frontalis, m. corrugator supercilii and m. procerius), and a significant increase in PPT was observed in both groups after treatment. The increase in PPT values suggested that these physiotherapy approaches may increase the mechanical pain threshold and decrease muscle sensitization in patients with migraine [35].

In our study, pain algometry measurements were performed on the anterior scalene, trapezius, suboccipital, temporalis, levator scapulae and SCM muscles from the right and left sides. Pain algometry parameters were compared before and after treatment in both groups. In the CTM and MLD groups, the pain threshold values increased, whereas the pain frequency and intensity decreased after treatment. This clinical improvement may be attributed to the higher frequency and duration of therapy sessions used in this study than in other studies. An increase in the pain threshold is associated not only with a reduction in pain intensity but also with greater ease of movement in daily life for migraine patients. Additionally, a reduction in pain frequency and intensity indicates significant long-term improvements in the quality of life of migraine patients. An increase in the pain threshold and a decrease in pain intensity further support the effectiveness of these treatment methods in the management of migraines. A study examining the effects of Ischemic Compression Myofascial Trigger Points therapy in 31 patients with episodic migraine revealed that while the tone and stiffness of the upper trapezius muscle decreased, pain in the shoulder and neck muscles was relieved, and the frequency and duration of headaches decreased significantly [36].

Migraine patients are often administered pharmacological treatments to manage and prevent attacks. The prescription rates for migraine and women with migraine have increased over the past 20 years [37]. However, some patients turn to non-pharmacological solutions due to gastrointestinal or cardiovascular disorders caused by pharmacological treatments, as well as medical contraindications such as pregnancy and breastfeeding [38, 39]. In a randomized controlled trial investigating the effectiveness of MLD and traditional massage in migraine patients, 64 migraine patients with and without aura were included, and the groups were divided into three groups: the MLD group (n = 21), traditional massage group (n = 21), and control group (n = 22). The treatment sessions lasted 30 min and were performed once a week for eight weeks. After the treatment, a decrease in pain frequency was observed in both the MLD and traditional massage groups compared to the control group. The use of medication for migraine pain decreased only in the MLD group [40]. Longo* et al*. [41] investigated the effectiveness of MLD in individuals with chronic tension-type headaches and reported that 95% of the participants experienced a sense of well-being and muscle relaxation immediately after treatment. The pain, although not significant, after treatment was lower than that before treatment.

In our study, the number of painful days, medication use and migraine pain severity decreased in the MLD and CTM groups after six weeks of treatment. When the duration of pain was analyzed, it was found to be shorter in the MLD group than in the CTM group. These findings suggested that MLD and CTM help patients manage pain more effectively and reduce the need for pharmacological treatment by reducing pain intensity and attack frequency.

Although migraine does not cause physical abnormalities, recurrent pain, and accompanying symptoms frequently affect the ability of patients to function normally. Attacks occurring at different times greatly reduce the adaptation of individuals to daily life and quality of life [42]. Clinically, significant improvements in quality of life are found as the frequency of headaches decreases, and these improvements are important indicators of the successfulness of the treatment process. A decrease in the frequency of headaches as a result of any treatment protocol applied may allow patients to participate more in activities of daily living and to improve their general health status [43, 44]. In a study by Celenay *et al*. [45], 16 women with migraine were randomly divided into two groups: CTM (CTM+ education (Ed) program, n = 8) and control groups (only Ed program, n = 8). The intervention consisted of one session of education and 12 sessions of CTM over a four-week period. After the intervention, the frequency, duration and severity of migraine decreased in the CTM group; associated symptoms such as nausea, photophobia and phonophobia were also significantly alleviated. In addition, medication use and disability levels decreased, and quality of life improved. These results suggest that connective tissue massage can improve quality of life in women with migraine and can be considered as a non-pharmacologic complementary treatment [45].

In this study, CTM and MLD improved vitality, pain, social functioning, mental health and general health perception, whereas MLD also had a stronger effect on emotional and physical role functions. Accordingly, both methods improved the quality of life of migraine patients.

## 5. Strengths and limitations

This is the first study in Turkey to examine the effectiveness of MLD in migraineurs. Although many studies have compared the effects of CTM and MLD in different diseases, this is the first study directly comparing the effectiveness of these two manual therapeutic methods on migraine. Our study showed that along with pharmacological treatment or an independent approach, these manual therapeutic methods can offer a viable treatment option without any side effects. In this context, the findings obtained are an important guide to better understanding the role of manual therapy in migraine treatment and guide clinical practice.

One main limitation of our study is that the findings are based on short-term results. Although the participants were followed up with a pain diary for 15 days after treatment, longer follow-up is needed to determine the long-term efficacy of MLD and CTM. Analyzing long-term responses to treatment may contribute to a comprehensive understanding of the clinical importance of these modalities. Moreover, the gender distribution of the individuals included in the study was not balanced, and given the sample size, it was not possible to determine the effects of variables such as age or gender on treatment. Additionally, migraine patients were not divided into subgroups according to their clinical migraine classification. This limited our ability to assess whether treatment responses differed according to specific migraine types. Despite these limitations, the results of our study support manual therapy methods a viable option along with or independent of pharmacological approaches in migraine treatment. Future studies should reinforce these findings with larger sample sizes, long-term follow-up, and grouping according to migraine subtypes.

## 6. Conclusions

In this study, MLD and CTM were effective in migraine treatment, supporting pain management by reducing medication use, and increasing the pain threshold. Additionally, while MLD significantly decreased pain duration, CTM was more effective in reducing neck pain and associated functional limitations. Additionally, both methods contributed significantly to improvements in quality of life. A reduction in pain duration, severity and medication use, along with the alleviation of neck pain, plays a crucial role in improving quality of life of migraineurs. Our findings were consistent with those reported in other studies. From a clinical perspective, non-invasive methods applied along with pharmacological treatment can improve pain management and enhance the quality of life of migraine patients. In this context, a personalized and holistic approach should be adopted in migraine treatment, integrating appropriate therapeutic approaches such as manual therapeutic techniques (*e.g.*, CTM and MLD) and patient education into treatment programs.
